# Supplementary material for: Interventions to Vaccinate Zero-Dose Children: A Narrative Review and Synthesis
Source: Viruses. 2023 Oct 14;15(10):2092. doi: 10.3390/v15102092 (PMC10612020; doi:10.3390/v15102092)
Supplement: Supplementary file 1 [file viruses-15-02092-s001.zip › Flow-Diagram-Zero Dose Manuscript 2023 10 12.pdf]

Figure S1: Flow diagram of included sources for narrative review for intervention strategies to reach zero-dose children (n = 27)

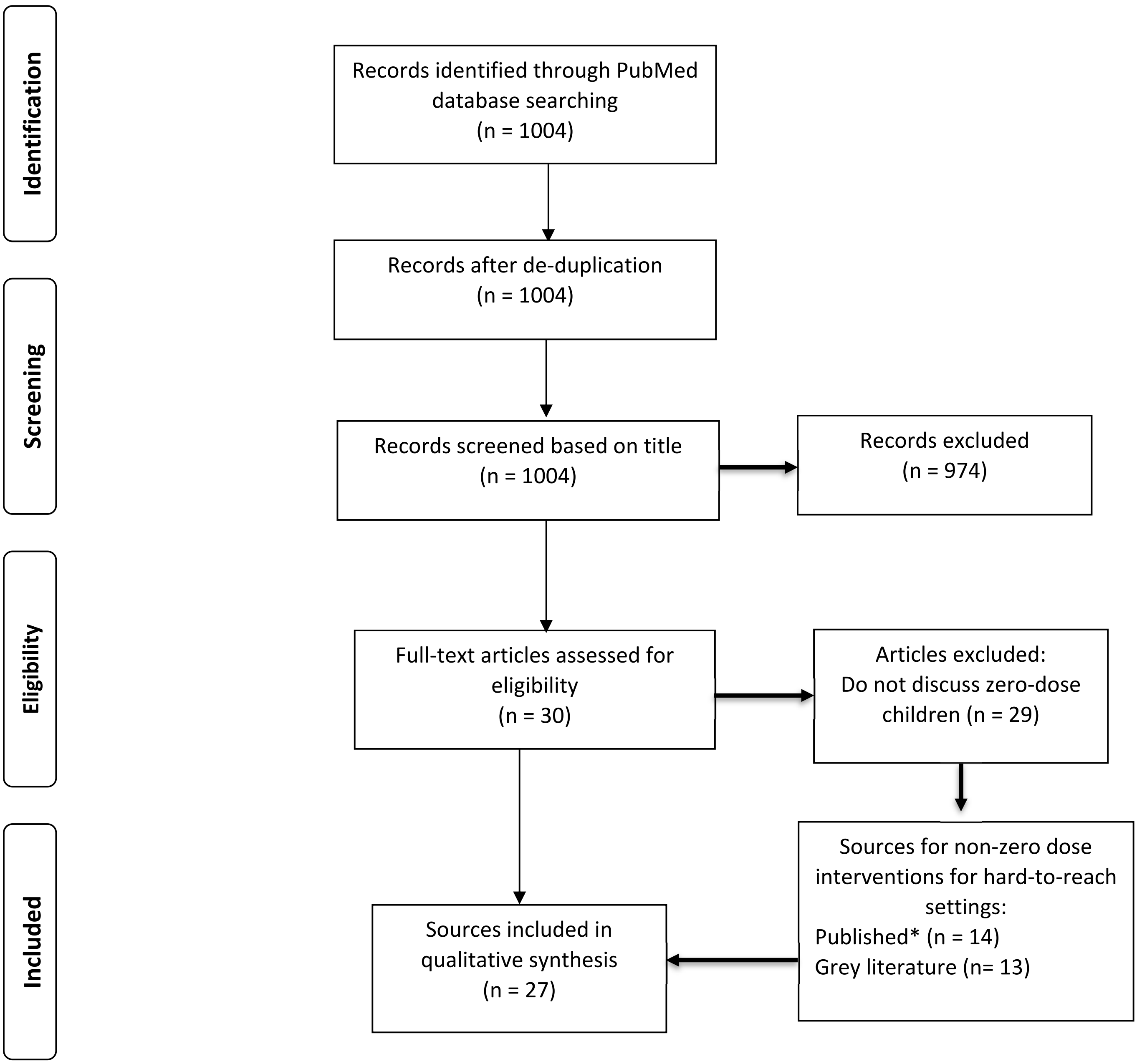

*Some of the articles excluded in the above step due to not discussing zero-dose children were now included in this step
