# Supplementary material for: Interventions to Vaccinate Zero-Dose Children: A Narrative Review and Synthesis
Source: Viruses. 2023 Oct 14;15(10):2092. doi: 10.3390/v15102092 (PMC10612020; doi:10.3390/v15102092)
Supplement: Supplementary file 1 [file viruses-15-02092-s001.zip › Key Informant Interview Guide.pdf]

START CENTER | STRATEGIC ANALYSIS, RESEARCH & TRAINING CENTER
Department of Global Health | University of Washington

Key Informant Interview Guide
Reaching Zero Dose Children

START Center

The START Center is a research consulting group at the University of Washington which leverages content expertise from across the University to provide high quality research and analytic support to the Bill & Melinda Gates Foundation, as well as to other public health decision makers, both globally and in the United States. The START Center also provides structured mentorship and training to University of Washington graduate research assistants.

Brief Background of Research

This project came from a request from the Global Delivery Programs (GDP) team at BMGF. The GDP team works with partners to ensure that low and middle-income countries can carry out high levels of routine and supplementary immunization activities that are effective, equitable, sustainable, and timely, focused on serving the most vulnerable. One of their priorities is to reach zero dose children, i.e., children who do not receive a single vaccine shot by 12 months of age. These account for ~75% of under-vaccinated children. Reaching these zero dose children is critical to both achieving equity and saving maximal number of lives. The specific aims for this project are included below.

- Review any existing data and evidence (both academic and grey literature) on why these communities may be underserved.

- Identify successful interventions to reach zero dose children and learn from other services or programs on finding and serving these difficult to reach populations (e.g., “last mile” efforts).

Interview Guide

This interview is expected to last between 40-45 minutes. All the answers that you/others provide will be kept anonymous. We will combine information obtained from you during this interview with that obtained from other interviews with other experts to provide an overall picture of what different stakeholders think about the subject matter. You can choose to abstain from answering any question that you would prefer not to answer. You can stop the interview at any time or ask us to clarify any questions that seem unclear to you. I also wanted to take your permission to record this discussion. This is only to ensure that our notes are complete. Only the small group of people working on this study will have access to the information you give us.

Do you have any questions for me?

Section A: Introductions – 5 minutes

The interviewer should provide a brief introduction of the START Center and RAs.

1. Can you describe how you are currently involved in activities related to immunization of zero dose children?

Section B: Understanding barriers to reaching zero dose children – 10 minutes

1. During our research, we found literature suggesting similar barriers for zero dose children and routine immunization (for example: community level barriers such as poor maternal knowledge, poor facility level organization, etc.). How are barriers for zero-dose children different from under-vaccination? What barriers to reaching zero-dose children are unique?
2. How do these barriers vary in different contexts like fragile/conflict, urban vs rural, given wide variation of contexts by country?

Section C: Understanding interventions to reaching zero dose children – 25-30 minutes

1. What types of interventions are you aware of for reaching zero-dose children specifically?
2. Could you share any examples of successful interventions on finding and reaching zero-dose children and missed communities through routine immunization channels and campaigns?
3. Thinking beyond routine immunization approaches, could you share any examples of interventions or programs from other health sectors on finding and serving the zero dose children?
    - Probe: Consider different contexts like fragile/conflict, urban vs rural, remote and rural populations, etc.
4. Is there any evidence on cost effectiveness and sustainability (ability for countries or partners to sustain results or activities long term, programmatically and financially)?

5. What barriers or challenges have these interventions encountered to date?
    - Probe: Are there particular gender-related barriers or considerations for such interventions that we should keep in mind while implementing them?
6. Finally, could you share any ideas or examples from some radically different sectors (e.g., finance/business, social media, consumer goods) that can be applicable here, which we can create parallels and take inspiration from to reach zero dose children?
7. Is there anything else you would like to share with us regarding this topic of zero-dose children? Are there any other resources, like websites, individuals, or organizations we should reach out to next?

Thank you so much for your time!
