# Supplementary material for: Interventions to Vaccinate Zero-Dose Children: A Narrative Review and Synthesis
Source: Viruses. 2023 Oct 14;15(10):2092. doi: 10.3390/v15102092 (PMC10612020; doi:10.3390/v15102092)
Supplement: Supplementary file 1 [file viruses-15-02092-s001.zip › KII Synthesis Template for manuscript 2023 09 26.pdf]

Reaching ZD Children_ KII Synthesis Memo Template   Feb 2022

Participant [Number]: [Participant name]

**Organization:**	
**Role:**	
**Barriers (include relevant quotes here)**	
**Proposed interventions from health sector (include relevant quotes here)**	
**Proposed interventions from non-health sector (include relevant quotes here)**	
**Any specific inputs by type of setting (conflict, rural, urban, etc.)**	
**Any other consideration/ recommendation for interventions to reach ZD children**	
