# Supplementary material for: Interventions to Vaccinate Zero-Dose Children: A Narrative Review and Synthesis
Source: Viruses. 2023 Oct 14;15(10):2092. doi: 10.3390/v15102092 (PMC10612020; doi:10.3390/v15102092)
Supplement: Supplementary file 1 [file viruses-15-02092-s001.zip › Supplemental Table S1.pdf]

Supplemental Table S1. A summary of the included sources for narrative review for intervention strategies to reach zero-dose children (n = 27)

Authors/name	Year	Country/region	Setting	Intervention domain[1]
Enkhtuya et al	2009	Mongolia	Rural/Urban	Health system strengthening
Pancholi et al/Village Reach	2020	Global	Rural/Urban	Health system strengthening
International Rescue Committee	2016	Global	Rural/Urban	Health Systems Strengthening
World Food Programme	2021	Timor-Leste	Conflict	Health Systems Strengthening
Bunn et al	2020	Sub-Saharan Africa	Rural/Urban	Community Engagement
Hossain et al	2021	Pakistan	Rural/Urban	Health System Strengthening
Uddin	2010	Bangladesh	Urban	Health Systems Strengthening
Hossain et al	2019	India	Rural	Community engagement
Dadari et al.	2021	Global	Urban	Health Systems Strengthening and Community Engagement
Utazi et al.	2020	Nigeria	Rural/Urban	Health System Strengthening
Arambepola et al.[2]	2021	Zambia	Rural	Technological innovations
Nsubuga	2019	Uganda	Rural	Health System Strengthening
JSI	2022	Pakistan	Rural/Urban	Health Systems Strengthening
GPEI	2017	Afghanistan	Rural	Community Engagement
Sturgis	2015	India	Urban	Community Engagement
Tsega	2018	Global	Rural	Health Systems Strengthening

1 Organized according to the three predominant intervention domains identified in our review: (1) community engagement, (2) health systems strengthening and integration, (3) technological innovations, noting that two additional categories include: (4) >1 of the 3 intervention domains or (5) other than the three intervention domains.

2 This is the only relevant source identified via our initial search strategy limiting published articles to interventions relevant for zero-dose children only

Family Folders	2012	Ethiopia	Rural	Health Systems Strengthening
Schelling	2007	Chad	Rural	Community Engagement
Apolitical	2017	Tanzania	Rural	Health Systems Strengthening
Tech Mahindra	2021	India	Rural	Health Systems Strengthening and Technological Innovations
JSI	2020	Tanzania	Rural	Health Systems Strengthening and Technological Innovations
Peyraud et al	2019	Global	Rural/Urban	Technological innovations
WHO	2017	Global	Rural/Urban	Technological innovations
Gavi	2022	Ghana	Rural	Technological innovations
ReliefWeb	2018	Syria	Conflict	Health Systems Strengthening and Technological innovations
WHO	2022	Global	Urban/Rural	Health Systems Strengthening and Technological innovations
Terry	2018	Global	Conflict	Health Systems Strengthening and Technological innovations
